# Prevalence and risk factors for trypanosome infection in cattle from communities surrounding the Murchison Falls National Park, Uganda

**DOI:** 10.1186/s13071-021-04987-w

**Published:** 2021-10-07

**Authors:** Daniel Kizza, Michael Ocaido, Anthony Mugisha, Rose Azuba, Sarah Nalule, Howard Onyuth, Simon Peter Musinguzi, Rodney Okwasiimire, Charles Waiswa

**Affiliations:** 1 Department of Livestock and Industrial Resources, College of Veterinary Medicine Animal Resources and Biosecurity, Makerere University, Kampala, Uganda; 2 Department of Wildlife, Aquatic and Animal Resources College of Veterinary Medicine Animal Resources, Biosecurity Makerere University, Kampala, Uganda; 3 Department of Agriculture, Faculty of Vocational studies, Kyambogo University, P.0 Box 1 Kyambogo, Kampala, Uganda; 4 Central Diagnostic Laboratory, College of Veterinary Medicine Animal Resources and Biosecurity, Makerere University, Kampala, Uganda; 5 Department of Veterinary Pharmacy, Clinical and Comparative Medicine, College of Veterinary Medicine Animal Resources and Biosecurity, Makerere University, Kampala, Uganda

**Keywords:** ITS-PCR *T*. *vivax*, *T*. *congolense*, Risk factors, Prevalence

## Abstract

**Background:**

Bovine trypanosomosis transmitted by tsetse flies is a major constraint to cattle health and productivity in all sub-Saharan countries, including Uganda. The objectives of this study were to determine the prevalence of bovine trypanosomosis and identify its associated risk factors and the species of trypanosomes associated with the disease.

**Methodology:**

A cross-sectional study was conducted around Murchison Falls National Park, Uganda from January 2020 to April 2020. Trypanosomes were detected in blood samples by PCR analysis targeting the internal transcribed spacer 1 (ITS-PCR assays), and trypanosomes in positive blood samples were sequenced.

**Results:**

Of 460 blood samples collected and tested, 136 (29.6%) were positive for trypanosome infections and 324 (70.4%) were negative. The overall trypanosome prevalence was 29.6% (95% confidence interval 25.4–33.8%), attributed to three trypanosome species. Of these three species, *Trypanosoma vivax* was the most prevalent (*n* = 130, 28.3%) while the others were detected as mixed infections: *T*. *vivax* + *Trypanosoma* *congolense* (*n* = 2, 0.4%) and *T*. *vivax* + *Trypanosoma* *evansi* (*n* = 1, 0.2%). There were significant differences in trypanosome prevalence according to sex (*χ*^2^ = 62, *df* = 1, *P* < 0.05), age (*χ*^2^ = 6.28, *df* = 2, *P* = 0.0043) and cattle breed (*χ*^2^ = 10.61, *df* = 1, *P* = 0.001).

**Conclusions:**

Trypanosomosis remains a major limitation to cattle production around Murchison Falls National Park and interventions are urgently needed. In our study, the prevalence of trypanosome infections was high, with *T*. *vivax* identified as the most prevalent species. Age, sex and breed of cattle were risk factors for trypanosome infection.

**Graphical Abstract:**

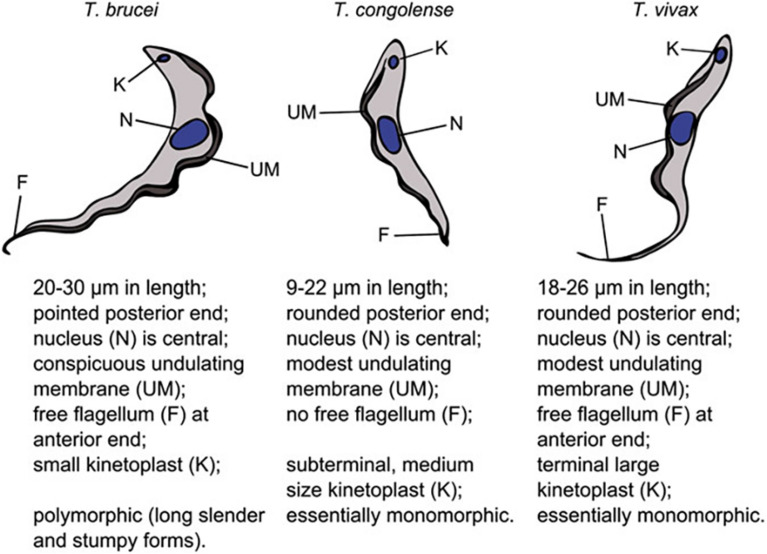

## Background

Trypanosomosis is one of the most important parasitic diseases in sub-Saharan Africa, constraining productive livestock farming, impeding economic development and causing a huge toll on human health [1, 2]. The most important trypanosomes affecting livestock are *Trypanosoma brucei brucei*, *Trypanosoma congolense* and *Trypanosoma* *vivax* in cattle, *Trypanosoma* *evansi* in horses, camels, water buffalos and cattle and *Trypanosoma* *equiperdium* in horses and donkeys [3]. In Uganda, *T*. *brucei brucei*, *T*. *congolense* and *T*. *vivax* are the main species of economic importance that cause bovine trypanosomosis transmitted by several species of tsetse flies (*Glossina* spp.) [4]. Human African trypanosomiasis in Uganda is mainly caused by two trypanosome subspecies: *T. brucei gambiense* in the north-western part of the country and *T. brucei rhodesiense* in south-eastern and north-eastern parts [5]. Cattle and wild animals are known reservoirs for *T*. *b*. *rhodesiense* [6–8].

The several different parasite species that cause bovine trypanosomosis affects the epidemiology, severity and management of the disease [9]. Accurate detection and identification of the different parasite species using PCR-based tools is crucial in the management and control of bovine trypanosomosis [10]. Analysis using PCR technique is based on the gene that encodes a small ribosomal subunit to identify and differentiate the most clinically important African trypanosome species and subspecies.

Determining the prevalence and identifying the trypanosome species of importance in a geographical region is essential for understanding epidemiology of the disease, especially zoonotic sleeping sickness. Furthermore, sleeping sickness remains an important public health challenge in Uganda, and identification of the drivers of its persistence in certain areas, including the role of wild animals and cattle reservoirs and identification of silent disease carriers, among others, needs urgent attention [11]. Information on the species of trypanosomes circulating among livestock in pastoral and agro-pastoral communities surrounding Murchison Falls National Park is vital to guide trypanosomosis control intervention programs by several stakeholders. Therefore, the objectives of this study were twofold: to determine the prevalence and associated risk factors of bovine trypanosomosis and identify the genetic diversity within the trypanosomes detected in cattle from communities surrounding Murchison Falls National Park in Uganda.

## Methods

### Study design and area

A cross-sectional study was conducted from January to April 2020 in Buliisa district (02°11ʹN, 31°24ʹE), Uganda. Prior to conducting the study, a preliminary reconnaissance visit was done to the study area through the Coordinating Office for the Control of Trypanosomosis in Uganda (COCTU).

Buliisa district was selected due to its location in the cattle corridor and its proximity to Murchision Falls National Park. The socio-economic activities in the district include pastoralism, agro-pastoralism, fishing and subsistence agriculture. The district has a bimodal type of climate with two rainy seasons (March–May and August–November). The vegetation is classified into forest, savannah, grassland and swamp, with the area in forest vegetation including Budongo forest, while the savannah comprises perennial grasses, scattered trees and shrubs. Murchison Falls National Park and Bugungu Game Reserve contribute to grassland and woodland cover. The national population and housing census of 2014 by the Uganda Bureau of Statistics (UBOS) reported a population of 113,161 people and 34,800 heads of cattle in the district. Buliisa sub-county was selected for the study as it had the largest cattle population in Buliisa district in that census and was the only sub-county in the district where people, livestock and wildlife share the same environment. Animals sampled in the study were selected from three villages in two parishes: Kataleba village in Bugaana parish, and Kabolwa and Kijanji villages in Kigoya Parish.

### Study animals and sample size calculation

The target population was all cattle-keeping farming households in Bugaana and Kigoya parishes in Buliisa sub-county. The sampling framework consisted of a list of all cattle-keeping households in the Bugaana and Kigoya parishes provided by the sub-county veterinary staff. The respective sample size of respondents was drawn from the list of cattle-keeping households in a parish by simple random sampling using Microsoft Excel® 2010 (Microsoft Corp., Redmond, WA, USA). The farmers agreed to participate in the study by signing consent forms that were provided to them prior to the collection of blood samples from their animals.

Animals sampled in this study belonged to the following breeds: local Zebu, Boran, crosses of Zebu and Boran and crosses of Zebu and Friesian. Information on age category (calf, heifer, steer or adult) and sex of the animals was also captured. Animals were grouped according to their body condition score into three categories (thin, borderline or moderate) based on the rib appearance and dorsal spines [12]. Specifically, animals were classified as thin when they showed no or very little fat deposition and there was muscle loss in the hind quarter; borderline when the fore ribs were noticeable and there was minimal muscle loss in the hind quarter; and moderate when all of the ribs were not visible to the naked eye and they were covered by muscle.

The minimum sample size determined was 388 animals, at a 95% confidence level, 5% precision level and an estimated proportion of trypanosomosis prevalence of 50% [13]. A total of 460 animals of both sexes, local and crossbreed, and of different ages were randomly sampled in the study.

### Sampling

Trained field veterinarians collected the blood samples from animals constrained in community crushes by puncturing the marginal ear vein with a lancet and collecting the blood into two micro-hematocrit tubes. Blood from the micro-hematocrit tubes was applied onto a designated sample area of Whiteman® FTA® classic cards taking precaution to avoid cross-contamination [1, 14]. The FTA® cards were labeled according to the animals’ name, breed, sex and age spotted, and then air dried. Later, the spotted FTA cards were packed in foil pouches containing silica gel desiccants and transported to the Central diagnostic Laboratory (CDL) College of Veterinary Medicine, Animals Resources and Biosecurity, Makerere University for PCR analysis.

### DNA extraction and PCR testing

DNA was extracted from discs punched out of the sample Flinders Technology Associate (FTA®) cards (Whatman® International Ltd., Maidstone, UK) following procedures previously described [15–17]. Briefly, DNA was extracted from FTA® cards by punching out sample discs with a Harris 3.0-mm micro punch. A total of five discs were punched from each sample area on the FTA® card into 1.5-ml microfuge tubes. The micro punch was cleaned by spraying with 70% ethanol, and at least five discs were punched out from a clean filter paper. To each microfuge tube (containing sample discs), 1000 µl of FTA purification reagent were added followed by incubation for 20 min at room temperature. This step was repeated before adding 1000 µl of TE buffer followed by incubation at room temperature for 15 min. This step that was also repeated. The sample discs were then transferred to a new microfuge tube and allowed to dry by incubation at 37 ºC for 45 min. A 100-μl volume of chelex 100 resin solution (5% chelex, w/v) was then added to the dried sample discs followed by heating at 90 °C for 30 min on a thermal block. The microfuge tubes were then briefly centrifuged and the eluate (without chelex) was transferred to a fresh 1.5-ml microfuge tube and frozen at − 20 ºC before analysis by PCR.

The samples were screened by PCR with primers targeting the internal transcribed spacer 1 (ITS1) of various trypanosome species: ITS1 CF (5′-CCGGAAGTTCACCGATATTG-3′) and ITS1 BR (5′-TTGCTGCGTTCTTCAACGAA-3′), as previously described [18, 19]. The ITS-PCR assays were carried out in 25-µl volumes containing 0.5 µM of each ITS1 CF and BR primers (Biolegio B.V, Nijmegen, The Netherlands), 12.5 µl of 2× Go Taq® Master mix (Promega Corp., Madison, WI, USA) and 5 µl of nuclease-free water (Promega Corp.), with 5 µl of the extracted DNA as template. Amplification was performed on a MultiGene™ TC9600-G thermal cycler (Labnet Inc., Edison, NJ, USA) as follows: an initial step at 94 °C, 5 min; then 94 °C/40 s, 58 °C/40 s, 72 °C/90 s for 35 cycles; with a final extension at 72 °C, 5 min .

The PCR amplification products together with a 100-bp standard molecular ladder (Thermo Fisher Scientific, Waltham, MA, USA) were electrophoresed in 1× TBE buffer on 1.5% agarose (Thermo Fisher Scientific) gels stained with ethidium bromide (Thermo Fisher Scientific). Gels were then visualized on a UV trans-illuminator (Syngene, Fredrick, MD, USA).

### Sequencing of PCR products

PCR products from the *Trypanozoon* sp.-positive sample, all the *T*. *congolense*-positive samples and 20 randomly selected *T*. *vivax* samples were sequenced in both directions by a commercial company. According to the protocol provided, PCR products were cleaned up with an ExoSAP-IT™ PCR Product Cleanup kit (Applied Biosystems™, Thermo Fisher Scientific) and sequenced using the BrilliantDye™ Terminator Cycle Sequencing Kit V3.1 (NimaGen B.V., Nijmegen, The Netherlands), following the manufacturers’ instructions. The labeled products were cleaned with the ZR-96 DNA Sequencing Clean-up Kit (Zymo Research, Irvine, CA, USA) and injected on to the Applied Biosystems ABI 3500XL Genetic Analyzer with a 50-cm array, using POP-7™ Polymer for 3500/3500xL Genetic Analyzers (Applied Biosystems™).

Sequence chromatogram analysis, manual clean-up, assembly and generation of census sequences were performed using the Bio Edit Sequence Alignment Editor version 7.2.5 [20]. The basic local alignment search tool (BLAST) [21] was used to identify homologous sequences in GenBank to those obtained in this study.

### Phylogenetic analysis

Multiple sequence alignment (MSA) of ITS1 sequences from this study and those obtained from GenBank was performed using the Molecular Evolutionary Genetics Analysis (MEGA X) software suite [22]. Phylogenetic analysis (Fig. 2) was performed using the neighbor-joining method [23]. The bootstrap test with 1000 replicates was performed to assess the robustness of the resultant cladogram.

### Data analysis

Information on name, sex, body condition score, age, breed and place of origin (village and parish) was entered into Microsoft Excel® 2010 and then exported to SPSS version 20 software (IBM Corp., Armonk, NY, USA). The chi-square test was used to determine the association between trypanosome infection rates and the risk factors. During the analyses, the confidence interval (CI) level was 95% and *P* < 0.05 was considered to indicate significance.

### Results and discussion

**Fig. 1:**
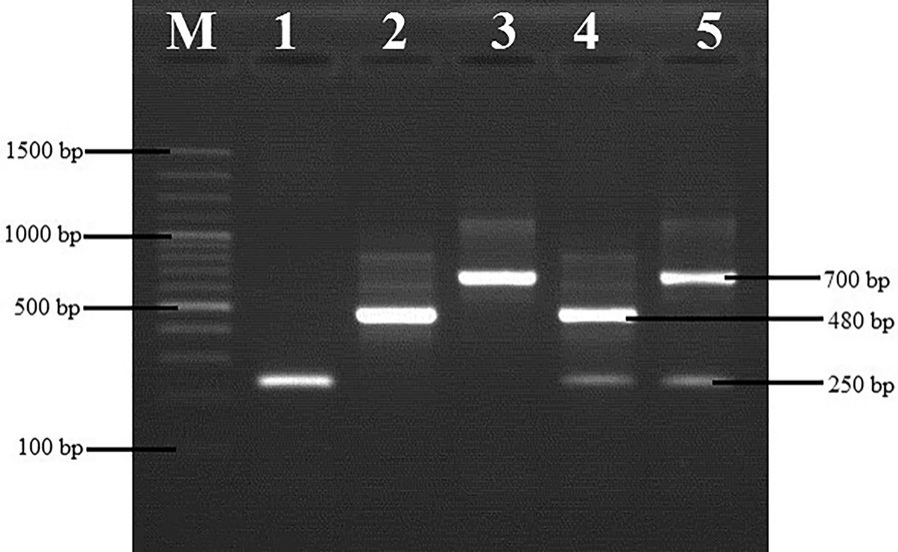
Representative agarose gel electrophoresis image of the ITS1 PCR products, showing the *Trypanosoma* spp. detected in cattle from Buliisa sub-county, Uganda. Lanes:* M* 100-bp DNA ladder, *T*. *vivax* (250 bp),* 2* *T*. *evansi* (480 bp),* 3* *T*. *congolense* (700 bp),* 4* *T*. *vivax* + *T*. *evansi* (mixed infection),* 5 T*. *vivax* + *T*. *congolense* (mixed infection)

The majority of cattle sampled were adult female crossbreeds (Table 1). Of the 460 samples that were tested, 136 (29.6%) were positive for trypanosome infection and 324 (70.4%) were negative, indicating an overall prevalence of trypanosome infection of 29.6% (95% CI 25.4–33.8%). *Trypanosoma vivax* (*n* = 130, 28.3%) was the most prevalent species and two mixed infection types [*T*. *vivax* + *T*. *congolense* (*n* = 2, 0.4%) and *T*. *vivax* + *T*. (*Trypanozoon*) sp. (*n* = 1.0, 0.2%)] were detected during the analysis (Table 2). A representative gel image of the results obtained is shown in Fig. 1.

**Table 1:** Description of studied cattle population (*n* = 460) from Buliisa sub-county, Uganda

Variable	*n* (%)
Age	
Adult > 36 months	212 (46.1)
Heifer/steers 12–30 months	137 (29.8)
Calves < 12 months	111 (24.1)
Sex	
Male	73 (15.9)
Female	387 (84.1)
Breed	
Local	157 (34.1)
Crossbreed	303 (65.9)

**Table 2:** Prevalence of trypanosome infection in cattle from Buliisa sub-county, Uganda

Species	No. of positives	Prevalence (%)	*χ*^2^; *df* (*P*-value)
*T*rypanosoma *evansi*	1	0.2	485; *df* = 4 (*P* < 0.05*)
*T*. *congolense*	2	0.4	
*T.* *vivax*	130	28.3	
*T*. *vivax* + *T*. *evansi*	1	0.2	
*T*. *vivax* + *T*. *congolense*	2	0.4	

*Denotes statistical significance at the 5% level

Sequence analysis of the ITS1 sequences generated in this study revealed lengths ranging from 192 to 254 bases for *T*. *vivax*, 313 bases for *T*. (*Trypanozoon*) sp. and 189 bases for *T*. *congolense*. The *T*. *vivax* ITS1 sequences (GenBank: MZ151473, MZ151469, MZ151466, MZ151477, MZ151476, MZ151472, MZ151475, MZ151471, MZ151470, MZ151467, MZ151474 and MZ151479) were homologous to several other ITS1 sequences available in GenBank. Furthermore, the BLAST results showing query length ranging from 209 to 251 bases and homology of 97.7–98.0% (GenBank: JX910375.1, MK880188.1, MN213748.1, MH247152.2, LC589626.1 and AB742529.1) were considered for phylogenetic analysis. The ITS1 sequence from *T*. (*Trypanozoon*) sp*.* (GenBank: MZ151468) was homologous to several *T*. *evansi* sequences available in GenBank, and BLAST results with query length ranging from 450 to 532 bases and homology of 97.4–98.9% (GenBank: MK696284.1, KY114581.1, MW272928.1, FJ712715.1, and MT539999.1) were considered for phylogenetic analysis. The *T*. *congolense*-positive sample (GenBank: MZ151478) was homologous to several other *T*. *congolense* sequences available in GenBank, and BLAST results with query length ranging from 241 to 785 bases and homologies between 89.5 and 99.2% (GenBank: MK132071.1, MG255216.1, AB742531.1, MK756202.1, and MH796911.1) were selected for phylogenetic analysis.

The overall prevalence of trypanosome infection in the study area (29.4%) was higher than the national mean prevalence of 14.28% (95% CI 10.39–18.67) [24], but lower than the prevalence of 41% reported in Nwoya and Amuru districts in a similar study [1] which also used ITS-PCR. Another study in south-eastern Uganda reported a lower prevalence of 15.3% [15]. These differences in prevalence can be attributed to numerous factors, including differences in vegetation types and seasons when the studies were conducted; these factors are known to affect the tsetse populations and ultimately the prevalence of trypanosome infections.

*Trypanosoma vivax* was the most common trypanosome species in the study area, representing 95.6% of the infections (Table 2). This finding is consistent with previous studies conducted in Uganda [4, 6]. Unlike other trypanosomes, this species is also transmitted mechanically by other biting flies [25], the mostt plausible explanation for the predominance of* T. vivax*. Another possible explanation might be that *T*. *vivax* has a shorter life-cycle in the tsetse fly proboscis [26, 27] and a quick multiplication of parasitemia in the host which could lead to high detection in cattle [28]. The observed predominance of *T*. *vivax* in terms of prevalence compared to other trypanosome species may also be due to the high presence of the the testse fly species *Glossina fuscipes*, which is known to transmit *T*. *vivax* compared to other *Glossina* spp. [29, 30]. Our results align well with those of a previous study [31] that reported a high abundance of *Glossina f. fuscipes* in the study area. The prevalence of other trypanosome species (*T*. *congolense*, *T*. *vivax* + *T*. *evansi* and *T*. *congolense* + *T*. *vivax*) were comparably low relative to that of *T*. *vivax*.

In this study, female cattle were significantly more infected (*χ*^2^ = 62, *df* = 1, *P* < 0.05) than male cattle (Table 3); this difference may be explained by the fact that farmers tend to keep fewer males in their herds than females. In addition, few males were sampled in the present study compared with females. A study in South-East Uganda showed a significant difference between trypanosome infection and the sex of the animals [32]. In contrast, a study in Tanzania revealed that sex and breed were not significant risk factors affecting trypanosome infection [33].

**Table 3:** Prevalence of trypanosome infection according to sex, breed, age and body condition score in Bugaana and Kigoya parishes

Variable	No. of examined cattle	No. of infected cattle	Prevalence (%)	χ^2^; *df *(*P*-value)
Sex				62; *df* = 1 *(P* < 0.05*)
Female	387	114	84	
Male	73	22	16	
Age				6.28; *df* = 2 (*P* = 0.043*)
Adult	212	59	43.38	
Heifers/steers	137	37	27.20	
Calves	111	40	29.41	
Body condition score				2.4; *df* = 2 (*P* = 0.301)
Thin	142	48	35.3	
Borderline	143	37	27.2	
Moderate	175	51	37.5	
Breed				10.61; *df* = 1 (*P* = 0.001*)
Local breed	157	49	36	
Cross breed	303	87	64	

* Denotes statistical significance at the 5% level

Regarding age as a risk factor, we observed a significant difference between age and prevalence of trypanosome infection (*χ*^2^ = 6.28, *df* = 2, *P* = 0.0043) despite cattle of different ages in the study area having been subjected to the same vector risk exposure (Table 3). The difference can be attributed to the higher attraction of tsetse flies to mature—hence bigger—animals than to calves [34]. In addition, adult cows produce more odor plumes than calves, resulting in an increased attraction of tsetse flies for the former [35]. Another plausible explanation may be that calves were being grazed in areas close to households with less risk of tsetse infestation compared to the high-risk distant grazing lands where heifers/steers graze.

Whereas the body condition score of animals is always associated with their health and nutritional status, this study did not find significant difference (*χ*^2^ = 2.4, *df* = 2, *P* = 0.301) between trypanosome prevalence and body condition (Table 3). Animals in good body condition score have been found to be more resistant to trypanosome infection compared to those in poor body condition in the wet season as reported in Cameroon [36]. A likely explanation to our finding is that at the time of conducting this study, animals’ nutritional body reserves were not yet severely depleted and therefore no considerable variation in the body condition score was evident.

The results showed a significant difference between breed and trypanosome prevalence (*χ*^2^ = 10.61, *df* = 1, *P* = 0.001), with a higher prevalence observed in crossbred animals (Ankole/Friesians, Zebu/Friesians or Boran/ Friesians) compared to the predominantly local breeds (Ankole, Zebu, and Boran) (Table 3). Farmers were crossbreeding their local cattle with exotic breeds as an overall strategy to transition from a low-input/low-output system to a high-input/high-output system, as stated previously [37].

**Fig. 2:**
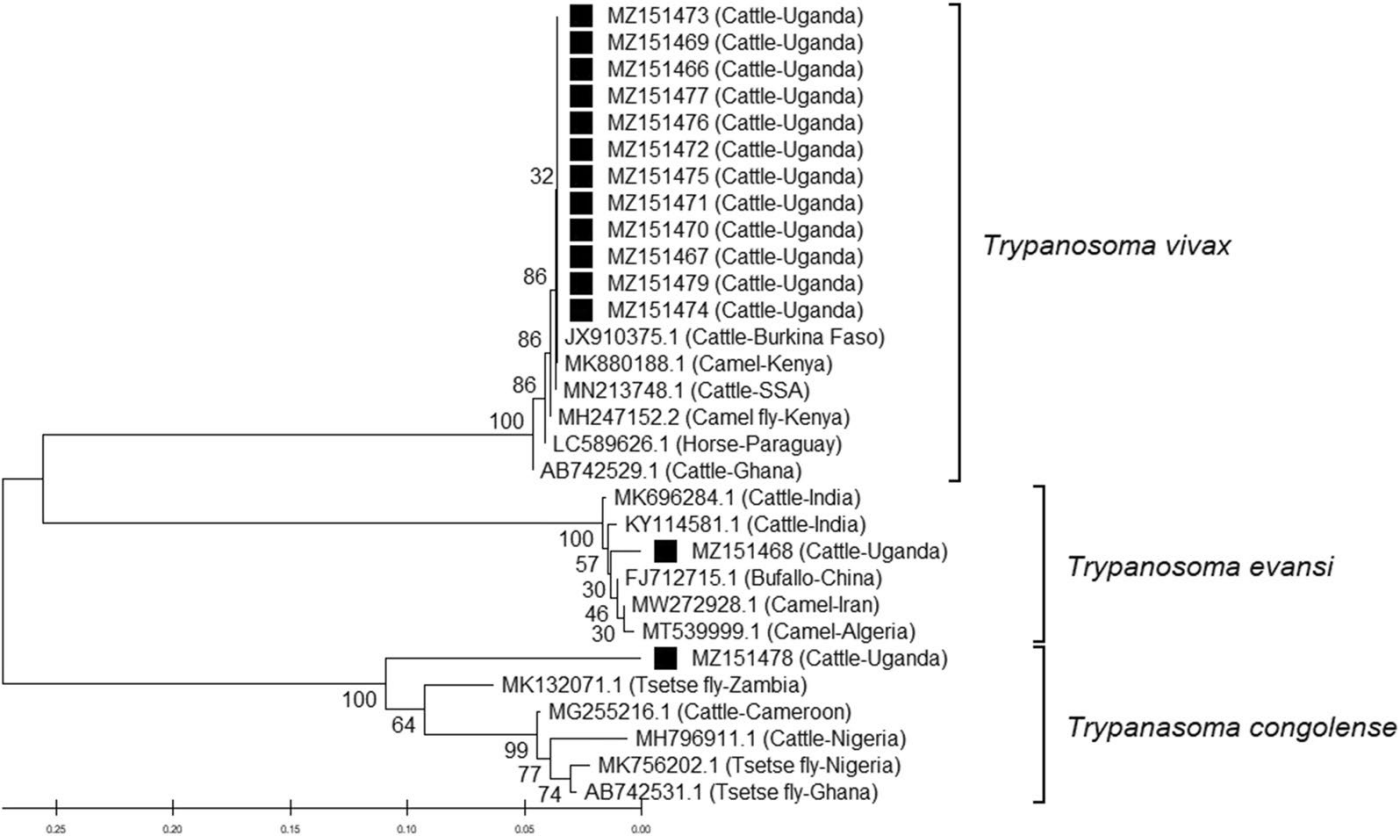
Phylogenetic tree based on partial ITS1 sequences from *Trypanosoma* spp. generated in this study and from GenBank. The tree was constructed using the neighbor-joining method. The branch length scale, shown below the tree, indicates the evolutionary distance computed based on the maximum composite likelihood method [38]; the unit is the number of base substitutions per site. The numbers at each node represent the percentage of bootstrap values based on 1000 replicates [39]

In this study we characterized the genetic diversity of trypanosomes from naturally infected cattle in Uganda. The phylogenetic reconstruction (Fig. 2) generated three major clades. The *T*. *vivax* detected in cattle in Buliisa sub-county was placed in a clade with sequences from East Africa, West Africa (Ghana, Burkina Faso) and South America (Paraguay). The phylogenetic analysis further placed *T*. *evansi* in a clade with strains from Asia (buffalo in China) and North Africa (camels in Algeria), while *T*. *congolense* was positioned in a clade with strains from Southern Africa and West Africa.

The present study has a number of limitations. The cross-sectional approach of the study did not allow us to collect blood samples from animals during both wet and dry seasons to evaluate the seasonal effect on infection prevalence. Also, due to limited resources, the study was conducted in one sub-county, and there was no entomological survey conducted to identify the major species of tsetse flies in the area.

## Conclusions

In conclusion, the results of this study confirm a high prevalence of trypanosome infection in cattle from Buliisa sub-county and that *T*. *vivax* is the predominant species in the area. In addition, the findings indicate that age, breed and sex of the animals are risk factors for trypanosome infection in cattle from Buliisa sub-county.

## Data Availability

Data supporting the conclusions of this article are available from the corresponding author on reasonable request. The sequences generated in this study were deposited in the GenBank under the accession numbers MZ151466 to MZ151479.

## Abbreviations

BLAST: Basic local alignment search tool
CDL: Central diagnostic laboratory
FTA: Flinders Technology Associates
ITS1: Internal transcribed spacer 1
MEGA: Molecular evolutionary genetics analysis
UBOS: Uganda Bureau of Statistics
